# IHR amendments and the “pandemic agreement” an Israeli perspective

**DOI:** 10.1186/s13584-025-00676-6

**Published:** 2025-03-13

**Authors:** Shelly Kamin-Friedman, Nadav Davidovitch, Hagai Levine, Dorit Nitzan

**Affiliations:** 1 The School of Public Health, Faculty of Social Welfare and Health Sciences, University of Haifa, Haifa, Israel; 2 Department of Health Policy and Management, Faculty of Health Sciences, Ben-Gurion University of the Negev, Beer Sheva, Israel; 3 Braun School of Public Health and Community Medicine, Faculty of Medicine, The Hebrew University of Jerusalem and Hadassah, Jerusalem, Israel; 4 Masters Program in Emergency Medicine, School of Public Health, Ben Gurion University of the Negev, Beer Sheva, Israel

**Keywords:** International health regulation (IHR), Pandemic agreement, World health organization (WHO), Equity, Solidarity, Vaccines

## Abstract

**Background:**

The 77th World Health Assembly in May 2024 agreed on several key amendments to the International Health Regulations (IHR) (2005), which are set to enhance global public health preparedness and response mechanisms. These amendments are part of a broader effort to integrate the lessons learned from the COVID-19 pandemic, seeking to create a more globally interconnected and rapid global response mechanism for future health crises, including a new Pandemic Agreement.

**Main body:**

Globally and in Israel, some voice their concern that the IHR amendments and the Pandemic Agreement could undermine a nation’s sovereign right to manage its public health response, infringe on national autonomy, or impose obligations such as sharing resources like diagnostics, medicines, technology, or vaccines, which could be seen as detrimental to national interests. This manuscript describes the IHR amendments and the ongoing work on the Pandemic Agreement. It explains how the documents do not undermine national sovereignty and highlights the moral and utilitarian justifications for Israeli support of these global legal documents. From a moral perspective, Israel should be committed to promoting the value of global public health and universal health coverage at both the international and regional levels. From a utilitarian perspective, provisions ensuring access to products and information will assist Israel in preparing for and protecting against health threats originating in neighboring countries and globally. Moreover, asking countries to be better ready may promote awareness and actions of public health services in Israel, which has long suffered from budgetary and health workforce constraints.

**Conclusion:**

Israel must work to promote the endorsement of the Pandemic Agreement and the IHR amendments, as they are essential documents for addressing public health threats without compromising national sovereignty.

## Background

The COVID-19 pandemic, which killed millions of people, has led the WHO Member States’ negotiations on the updating the International Regulations (IHR) (2005) and on a new “Pandemic Agreement”. The common aim was to strengthen the global prevention, preparedness, and response to future pandemics.

However, globally and in Israel there are some who voice their concern that the IHR amendments and the Pandemic Agreement could undermine nation’s sovereign right to manage its public health response, infringe on national autonomy or impose obligations such as sharing of resources like diagnostics, medicines, technology or vaccines, which could be seen as detrimental to national interests.

To address these concerns, the following article discusses the content of the agreed IHR amendments and the “Pandemic Agreement” draft, detailing the moral and utilitarian rationales for advancing these documents from the Israeli perspective.

The paper refers to the draft IHR (2005) and the amendments agreed on June 1, 2024 [1] and to the proposed “Pandemic Agreement” between the member states of the World Health Organization (WHO), dated April 22, 2024 [2] (hereinafter: “the agreement”).

## Main text

### The International Health Regulations (IHR) 2005

#### Historical perspective

In 1851, the first International Sanitary Conference was held in Paris, proposing globally agreed quarantine measures to prevent the spread of cholera, plague, and yellow fever. In 1951, the World Health Assembly (WHA) of the World Health Organization (WHO) adopted the International Sanitary Regulations (ISR). These regulations, the precursor to the International Health Regulations (IHR), aimed to control six major infectious diseases: cholera, plague, yellow fever, smallpox, relapsing fever, and typhus. In 1969, the ISR were renamed the International Regulations, focusing on the six diseases while aiming to minimize the interference with world trade and traffic. The 1973’s amendments primarily focused on refining the definitions and the notification processes for the diseases covered under regulations. They also introduced more precise requirements for the vaccination certificates needed for international travel, particularly concerning yellow fever and smallpox. The 1981 amendments covered only three diseases (yellow fever, plague, and cholera), which posed the most significant risk of international spread and had implications for global public health security. At that time, the global eradication of smallpox was achieved.

The further streamlining of the IHR and their eventual expansion to cover all public health emergencies of international concern came with the comprehensive revisions in 2005, motivated by global experiences including the SARS outbreak. These revisions marked a significant shift from managing specific diseases to a broader approach that included “all-hazards” and the “whole-of-society” approaches for any event that might pose a risk to international public health. All 194 member states of the WHO are bound by the IHR (2005). Additionally, two non-member states, totaling 196 State Parties joined: Liechtenstein and the Holy See. Both agreed to abide by the regulations despite not being WHO member states [3]. 

#### Objectives of the IHR moving from 2005 to 2024

The IHR (2005) aimed to ensure a more effective and coordinated global approach to managing public health emergencies of international concern (PHEIC), safeguarding public health, and strengthening global health security at all levels. It defined the rights and obligations of the State Parties concerning the prevention, preparedness, and response to public health threats with global implications.

According to the updated version dated June 1, 2024, the purpose of the IHR is “…to prevent, prepare for, protect against, control and provide a public health response to the international spread of disease in ways that are commensurate with and restricted to public health risk and which avoid unnecessary interference with international traffic and trade.” The term “disease,” as used in the amended IHR, refers to “an illness or medical condition, irrespective of origin or source, that presents or could present significant harm to humans.” Harm to human agents includes events of chemical, biological, nuclear, or environmental origin.

The revised IHR focuses on proactive risk management by emphasizing early detection of global health threats rather than implementing restrictions on the movement of people and goods after a threat has emerged. The IHR operates under the assumption that early prevention and detection can stop outbreaks from turning into epidemics, prevent natural disasters from causing chaos, and avert humanitarian disasters stemming from conflicts.

### Key provisions of the IHR (2005)

#### Notification and information sharing

Member states are committed to notifying the WHO “of all events which may constitute a public health emergency of international concern”. These events may include outbreaks of specific diseases such as Smallpox, Poliomyelitis, human influenza caused by a new subtype, and SARS. Additionally, member states must report on other diseases that may require preparedness measures, including cholera, Pneumonic Plague, Yellow Fever, and Viral Hemorrhagic Fevers (such as Ebola or West Nile Fever). States must also report any event with potential global health implications, even if the source is unknown. Reports of disease outbreaks or public health threats should include laboratory findings, information about the source and type of risk, the number of cases and deaths, conditions affecting disease spread, and the health measures employed.

#### Determination of a Public Health Emergency of International Concern (PHEIC)

The IHR (2005) gives the WHO Director-General (DG) the authority to declare a PHEIC. This decision is made based on the advice of the Emergency Committee, which comprises experts in relevant fields and operates under the regulations.

Upon the declaration of a PHEIC, the State Parties should respond promptly to the health threat according to the guidelines and recommendations issued by the WHO. They are obligated to strengthen public health surveillance, collaborate on containment and control strategies, and share timely information and genetic and biological materials related to the threat.

#### Member States preparedness for emergencies

The IHR (2005) established a framework for state parties to develop specific core capacities for emergency preparedness. These core capacities are as follows: (1) Establishing a surveillance system to monitor and gather data on potential public health threats and events, regularly. This includes detecting and assessing events based on defined criteria and ensuring timely and effective reporting to WHO; (2) Having a public health response plan that can mobilize necessary resources during emergencies. This involves having a trained workforce, established communication and coordination mechanisms, and access to a stockpile of medical supplies and equipment; (3) Health systems capable of responding to emergencies, including having the infrastructure and health workforce capable of managing and treating cases as needed; (4) Effective communication strategies to inform and educate the public about health threats and measures they can take to protect themselves, including travelers and communities at risk; (5) Maintaining the capacity to apply health measures at points of entry (PoE) to prevent the spread of health threats, which includes inspection services, isolation facilities, and appropriate medical services; (6) Available laboratory services to safely, timely, and accurately detect and diagnose, including the ability to ship samples to reference laboratories when domestic capabilities are exceeded; (7) Developing and maintaining a public health workforce capable of implementing IHR provisions; and (8) Adequate legal framework and financing mechanisms ready to support the national implementation of IHR provisions and maintain the required health capacities.

To track and improve the implementation of the IHR (2005) core capacities, the State Parties agreed on the IHR Monitoring and Evaluation Framework, which includes the following tools: (1) Each of the IHR State Parties has to report annually using the State Party Annual Reporting (SPAR). The reports are submitted to the WHO and include their status and progress in achieving and maintaining the core capacities required under the IHR. This self-assessment shared globally during the WHA and thereafter, helps monitor progress and identify gaps in preparedness and response capabilities; (2) The Joint External Evaluation (JEE) is a voluntary, collaborative process to assess a country’s capacity to prevent, detect, and respond to public health threats. The JEE involves a team of international experts working alongside national experts to evaluate capabilities across relevant technical areas, such as surveillance, laboratory systems, and emergency response; (3) Intra- and After-Action-Reviews (AAR and IAR) are conducted following a public health emergency to assess their responses and identify lessons learned. This process helps improve future readiness and response strategies by highlighting effective practices and areas needing improvement. A similar tool, the Intra Action Review (IAR), was developed and implemented during the COVID-19 pandemic to improve the ongoing response operations; (4) Simulation Exercises (SIMEX), table-top or field-based, are used to test the operational readiness of emergency response systems in a risk-free environment. They help identify weaknesses in emergency plans and communication systems, enhancing overall preparedness. The IHR (2005) has set the benchmark for preparedness, and its evaluation and monitoring, all geared towards a world that is better prepared and agile.

#### Recommendations for handling international emergencies

Based on the IHR (2005) in the event of a PHEIC, the WHO provides state parties with recommendations, such as on diagnostics, treatment, vaccination and public health and social measures, including, isolation and quarantine. These recommendations are crafted by the leading global experts based on scientific evidence and principles, data transparency, and accessibility to the recommended measures. The selected actions should be proportionate to the risk and minimize the impact on individual rights, international traffic, and trade. Member states may implement additional or alternative measures in response to public health threats, provided these measures are scientifically based and involve minimal disruption to international travel and human rights. They should explain to the WHO the basis for them taking different actions. WHO’s recommendations for member states in handling public health threats are not binding.

#### International travel and supply chains

The IHR extensively addresses the issue of cross-border travel and commerce during global health threats. Member states may require cargo inspections, medical examinations for travelers, vaccinations (subject to informed consent), or isolation upon entry into their territories, provided that the least restrictive measures necessary to prevent disease spread are taken. They are requested to explain the basis for activating restrictive measures to the WHO.

#### Cooperation

The IHR asks the State Parties to strive for cooperation in identifying and responding to global health risks, including technical, logistical and financial aspects.

### Key amendments to the IHR (2005)

The Working Group on Amendments to the IHR (WGIHR) was established in response to the WHO Executive Board (EB) Decision 150(3) and further defined by the WHA Decision WHA 75(9) in 2022. The WGIHR was tasked with reviewing and proposing changes to enhance global health security. The amendments are important for, among other things, ensuring equitable access to health products and guaranteeing the effective implementation of the IHR.

#### Ensuring equitable access to health products

One of the 2024 meaningful additions to the IHR (2005) is the focus on improving equitable access to medical products and resources during health emergencies. This aims to ensure that all countries, regardless of their economic status, have access to necessary medical supplies and technologies during a public health emergency. Member states commit to allocating financial resources for this purpose and supporting WHO’s coordination efforts. Additionally, according to the amended IHR, the WHO will assist member states, upon their request, in promoting access to health products through platforms that enable equitable and fair distribution.

#### Ensuring implementation of the regulations

The response to the COVID-19 pandemic led to the realization that effective global cooperation is impossible if member states cannot rely on each other to follow WHO guidelines [4]. Therefore, a committee responsible for implementing the regulations will be established in line with the proposed amendments to the IHR. This committee will assist member states with the technical, logistical, and financial aspects relevant to the implementation of the regulations, through fostering cooperation and consensus rather than through confrontation or punishment.

The IHR amendments are detailed in Table 1.

**Table 1:** Key amendments to the IHR (2005)

IHR (2005) Article	2005 Description	2024 Amendments/Additions	Implications/Comments
Article 1	Definitions, including health products	Expanded definitions to include therapeutics, diagnostics, and other health technologies.	Clarifies the scope of health products to adapt to modern health needs.
Article 4	Responsible authorities for implementing health measures	Establishment of National IHR Authorities to improve coordination.	Improves implementation and monitoring of IHR provisions within and among countries.
Article 5	Surveillance requirements for public health events	Strengthened surveillance with support for developing countries; periodic reviews.	Enhances global surveillance capabilities for rapid detection and response.
Article 6	Notification of public health events to WHO IHR focal point	Improved communication systems for timely notifications with enhanced specificity in the types of events that must be notified and the timeline for notification.	Improves the timelines and detail in the reporting of potential public health emergencies to WHO and facilitates quicker communication of health threats and the response to them.
Article 9	Other reports	Expanded to include non-state actors and private sector reports of public health risks.	Broadens the scope of surveillance and data collection, incorporating reports from various sources beyond national governments.
Article 12	Determination of a public health emergency of international concern	Introduced a definition of a “pandemic emergency” to trigger more effective international collaboration.	Enhances the global response to pandemics by setting clear criteria for action.
Article 13	Public health response capacities	WHO provides guidelines and support for capacity development, including logistics and operational support. States must respond effectively.	Enhances capacities and capabilities for readiness and coordination in health emergencies among states.
Article 16	Standing recommendations for health measures	WHO may issue recommendations for ongoing health measures.	Ensures consistent health measures for ongoing risks and threats.
Article 17	Criteria for recommendations	Clarified processes for issuing temporary recommendations and the criteria for their issuance.	Provides clearer guidance on how and when WHO issues temporary recommendations, enhancing transparency and compliance.
Article 18	Recommendations at points-of-entry	Revised to include measures related to new types of threats, such as antimicrobial resistance and new pathogens.	Updates health security measures at borders to adapt to evolving health threats, ensuring better containment of diseases.
Article 44	Collaboration and assistance	Increased obligations for collaboration and financial support during public health emergencies.	Ensuring more effective deployment of international resources during crises.
Article 55	Procedures for proposing amendments	Streamlined amendment proposal process to ensure timely updates.	Improves the adaptability of the IHR to future challenges with timely amendments.
New Article		Introduction of a definition and procedures for “pandemic emergencies” to trigger specific response mechanisms.	Enhances global response capabilities by providing clear criteria for declaring and managing pandemic emergencies.
New Article		Establishment of a Coordinating Financial Mechanism to support identification of, and access to, financing required for developing, strengthening, and maintaining core capacities.	Supports developing countries in building necessary public health infrastructure, ensuring equitable access to resources needed for pandemic preparedness.
New Article		Creation of National IHR Authorities to improve coordination of implementation of the Regulations within and among countries.	Facilitates better national implementation and compliance with IHR through designated national bodies, enhancing global health security collaboration.
New Article		Introduction of an article addressing the use of digital health tools and data privacy during health emergencies.	Recognizes the growing role of digital health technologies in managing public health and outlines standards for their use and data protection.

The package of amendments was adopted by consensus, i.e. not requiring a vote, by the Seventy-seventh World Health Assembly on 1 June 2024. In accordance with Article 59 of the IHR, the amendments will come into force 24 months after their notification by the Director-General to all States Parties. Under Articles 59 and 61 of the IHR, member states can object to new amendments or issue reservations, in which case those provisions will not enter into force with respect to that member state.

## The WHO “pandemic agreement”

### Background

During the global response to the COVID-19 pandemic, the WHO issued recommendations under its authority derived from the IHR (2005). However, member states implemented independent policies without coordination, including closing borders to the transit of people and trade, contrary to WHO recommendations. Many wealthy countries refused to support WHO platforms that were designed for sharing information and resources, including vaccines, and the WHO recommendations were not enforceable [5]. 

The Independent Panel for Pandemic Preparedness and Response was established following a resolution adopted at the 73rd WHA in 2020. This decision highlighted the need for an impartial and comprehensive review of the international health response to the COVID-19 pandemic. The panel’s mission was to evaluate the effectiveness of the WHO-coordinated response and make recommendations to improve global preparedness for future pandemics. The Panel, in its report that was published in May 2021, recommended the consideration of a global pandemic treaty, that will enable a stronger global governance, better resource allocation, and more effective cooperation among countries during health crises.

In May 2021 the WHA agreed to establish an Intergovernmental Negotiating Body (INB) to draft a pandemic treaty to enhance global preparedness for future pandemics. This body was tasked with developing a comprehensive framework to manage global health threats, underlining the necessity for more structured international collaboration on pandemic prevention, preparedness, and response [6]. 

### Objectives of the “pandemic agreement”

The objectives of the Pandemic Agreement are to prevent, prepare for and respond to pandemics according to the following principles: the sovereign right of states to legislate and implement legislation within their jurisdiction; protection of the right to dignity and other human rights, including the right to the highest attainable standard of health; adherence to the rules of international humanitarian law; equity; solidarity; and decision-making based on the best available scientific evidence.

### Key provisions of the “pandemic agreement”

The ongoing negotiations about the WHO Pandemic Agreement have focused on several important topics, headed by measures for pandemic prevention, preparedness, and response, equity in access to health products, and mechanisms for implementing agreement provisions.

#### Measures for pandemic prevention, preparedness, and response

Signatory countries are required to implement various measures to prevent pandemics. These measures include maintaining water sanitation, ensuring routine vaccinations, and managing risks in laboratories to prevent pathogen exposure. Additionally, countries commit to promoting the “One Health” approach, which recognizes the interconnection between humans, animals, and the environment in the development of pandemics. To enhance pandemic preparedness, countries commit to strengthening their health systems to ensure equitable access to emergency health services; promoting investment in a multidisciplinary workforce skilled in pandemic response; adopting a cooperative approach among various authorities and involving communities and social organizations; developing public awareness programs and enhancing health literacy.

Beyond national measures, signatory countries are expected to support pandemic prevention, preparedness, and response efforts globally, especially in developing countries. Assistance to other countries will involve the transfer of necessary technologies and the sharing of relevant skills, both technical and scientific, to help manage pandemics effectively.

#### Equity in access to health products

Countries commit to promoting global access to necessary health products during a pandemic by taking measures to reduce the gap between demand and supply. These measures include: urging manufacturers to increase the production of health products during a pandemic; demanding fair pricing from manufacturers developing health products with government funding; encouraging manufacturers to disclose relevant knowledge and temporarily waiving intellectual property rights. Moreover, under the agreement, the Pathogen Access and Benefit-Sharing System - PABS is established to ensure the rapid sharing of biological samples of pathogens with pandemic potential, and the global access to health products developed based on these samples.

It is agreed that at least 20% of the health products necessary during a pandemic will be allocated to the WHO (10% as a donation and 10% at an affordable price) to ensure their fair distribution. Additionally, each country will, to the best of its ability, allocate a portion of the health products it acquires for pandemic response to be used by countries in need.

#### Mechanisms for implementing agreement provisions

Under the agreement, a Coordinating Financial Mechanism will be established to aid countries lacking the necessary resources for implementing its provisions.

Additionally, a Global Supply Chain and Logistics Network will be established to enhance equitable, timely and affordable access to pandemic related health products.

The parties to the agreement are committed to resolving disputes among them through negotiation/mediation/ or through an ad hoc arbitration tribunal. A Conference of the Parties will oversee the implementation of the agreement and periodically review it.

The following Table 2 outlines the major issues in the Pandemic Agreement, their acceptance or debate status, and their associated pros and cons.

**Table 2:** Key provisions of the “pandemic agreement”

Topic	Agreed Upon	Still Under Debate	Pros	Cons
Definition and Declaration of a Pandemic	Basic framework agreed	Specific means and procedures	Establishes a clear global standard for pandemic response activation.	Differences in national perspectives can complicate timely consensus.
Integration with the IHR	Some alignment strategies	How both frameworks will coexist without overlap	Ensures comprehensive global health laws	Potential for jurisdictional confusion
Equity in Global Supply Chain	General commitment to equity	Mechanisms to ensure fair distribution of medical resources	Promotes fair access to diagnostics, medicines, vaccines and technologies	Intellectual property rights and economic implications for pharmaceutical companies and countries
One Health	A recognized approach	Specific strategies and implementations under this framework	Encourages integrated health strategies that recognize the interconnection of human, animal, and environmental health	Challenges in aligning policies and practices across various sectors and regions, due to differing priorities and resources.
Financial Mechanisms	Recognizing the need for financing	Specifics of new financial mechanisms and contributions	Ensures funded readiness and response capabilities	Disagreements on contributions.
Technology Transfer and Intellectual Property	Recognized importance	Non-voluntary provisions like waivers during emergencies	Accelerates access to crucial technologies	Some nations with strong pharmaceutical industries resist
Governance	Framework established	Specific roles and powers of the Governing Body	Creates a structured oversight body	Power dynamics could skew equity efforts

The provisions detailed above are in draft and negotiation stages.

The IHR and the proposed Pandemic Agreement serve complementary but distinct purposes in global health governance. While both aim to enhance international preparedness and response to health threats, they differ in scope, objectives, and focus.

The IHR address a broad spectrum of public health emergencies with international implications. Its’ primary goal is to prevent, detect, and respond to health threats, including infectious diseases, chemical hazards, and radiological events. It operates under an opt-out system, binding all WHO member states by default unless they explicitly choose to opt out.

In contrast, the proposed Pandemic Agreement is a more targeted framework, focusing exclusively on pandemic prevention, preparedness, and response. It is being developed to address gaps highlighted during the COVID-19 pandemic, particularly issues of equity, global coordination, and resource allocation. Unlike the IHR, which provides general guidelines, the Pandemic Agreement is expected to introduce specific mechanisms for ensuring equitable access to vaccines, therapeutics, and diagnostics, as well as shared financial and logistical responsibilities among nations. The Pandemic Agreement follows an opt-in model, requiring states to explicitly commit to its provisions [7]. 

## Opposition to the IHR and the pandemic agreement

The current versions of the IHR and the Pandemic Agreement have opponents who believe that these texts might harm the sovereignty of the state.

In April 2024, several Israeli Parliament members sent a letter to the Minister of Health, the Minister of Foreign Affairs, and the Prime Minister, calling “to stop the harm to the country’s sovereignty.” According to them, “Without prior parliamentary or governmental consent, the WHO will be granted broad powers. Thus, the organization could intervene in the domestic and foreign policies of member states without an official vote and without an appeal process” [8]. Conspiracy theories have circulated on social media claiming that the documents aim “to control countries’ decision-making, determine threats.the reasons for declaring a pandemic emergency will be expanded and not just based on data. This is a risk to the sovereignty of nations.“ [9].

The Israeli antagonism toward cooperation with the WHO and other international organizations intensified during the “Iron Swords” war partly due to the unwillingness of these organizations to demand the release of Israeli hostages or, at the very least, to ensure the delivery of medications to them. WHO’s unequivocal support for the Palestinian side of the conflict has raised concerns that, under the IHR or the Pandemic Agreement, Israel may in the future be required to take actions that contradict its security interests, including potential demands to cease military operations.

Concerns about harm to national sovereignty have also been raised by countries other than Israel, driven by mistrust in the WHO due to its perceived failures in managing the COVID-19 pandemic. This mistrust is rooted in claims that member states were informed late about the virus’s emergence in China. This delay in notification is viewed as a critical factor that hampered early containment efforts and allowed the virus to spread globally before adequate measures could be implemented [10]. It was also claimed that the WHO’s opposition to border closures resulted in a lack of timely action that could have mitigated the spread of the virus [11]; and that the organization failed to provide clear and up-to-date information to member states and issued inconsistent guidelines [12]. 

It should be noted in this context that on January 20, 2025, President-elect Trump signed an executive order announcing the United States’ intention to withdraw from its membership in the WHO, referencing, among other reasons, the mismanagement of the COVID-19 pandemic. The order states: “While withdrawal is in progress, the Secretary of State will cease negotiations on the WHO Pandemic Agreement and the amendments to the International Health Regulations, and actions taken to effectuate such agreement and amendments will have no binding force on the United States.“ [13].

Alongside the opposition to the amendments to the regulations and the agreement on the grounds of mistrust and the concerns that they may harm the sovereignty of member states, it has been argued that the current texts will not achieve the goals of cooperation, fairness, and solidarity during a health crisis. The arguments supporting this claim are as follows: states are permitted to take into account their national laws, capacities, and circumstances, and the documents use terms such as “promote,” “endeavor,” or “undertake,” which indicate a weak level of commitment from the countries [14]. Additionally, there is no mechanism to assess whether states are fulfilling their obligations [15], and no enforcement mechanisms are in place [16].

There is an inherent tension between the aim of fostering fairness and solidarity through the regulations and the agreement, and the overriding priority of each member state to safeguard its sovereign authority to make decisions that prioritize its own national interests.

States are obligated to ensure the well-being of their citizens, and their leaders face political, economic, and social pressures to allocate resources to meet the health needs of their populations and act in accordance with national interests. However, pandemics and other threats to global health present challenges that transcend geographical boundaries, emphasizing the necessity of cross-border collaboration. The willingness to share resources, such as knowledge, medications, and vaccines, is sometimes perceived as a risk to vital national assets, yet it is also an essential tool for preventing and addressing global crises. This tension requires a delicate balance between achieving national health goals and contributing to international cooperation that yields mutual benefits.

The current texts of the IHR and the Pandemic Agreement do not undermine the sovereignty of member states. The definitions section (Article 1) in the IHR clearly states that the WHO’s recommendations for countries’ responses to public health threats are not binding. Additionally, the right of states to legislate and implement laws according to national health policies is explicitly mentioned in Article 3. The Pandemic Agreement (Article 24) states that the WHO shall have no authority to require member states to undertake specific actions, such as banning the entry of travelers, mandating vaccination or diagnostic tests, or imposing quarantines. The Association of Schools of Public Health in the European Region (ASPHER) held in this context: “It is crucial to understand that the WHO’s authority comes from its member states. While WHO can provide advice and support, the Pandemic Agreement does not grant WHO the power to amend national laws, impose vaccine mandates, non-pharmaceutical interventions (NPIs), or lockdowns. Article 19 of the WHO Constitution ensures that international treaties or agreements cannot override the democratic processes or the parliaments of WHA members who sign the Pandemic Agreement.” [17].

The IHR and the Pandemic Agreement provide a shared global infrastructure designed to assist countries in dealing with health crises. This infrastructure will enable countries to jointly design strategies for addressing future health crises that align with the principles of fairness and solidarity. Commitment to these values will be achieved through mechanisms that ensure transparency leading to mutual trust, promote open dialogue, and support decision-making by professional and non-political entities.

## Support of the IHR amendments and the “pandemic Agreement”– an Israeli perspective

### Moral reasons

The right to health is a universal right. According to the International Covenant on Economic, Social and Cultural Rights of 1966, “The States Parties to the present Covenant recognize the right of everyone to the enjoyment of the highest attainable standard of physical and mental health” (Article 12.1). Israel ratified the covenant in 1991 and is therefore committed to its values.

Alongside Israel’s commitment to promoting global health, it also has a commitment to advancing health at the regional level. Israel has a relatively strong economy compared to its neighboring countries, and a better ability to cope with a health crisis. Israel should therefore take the lead in creating collaborations aimed at promoting the health of the entire region [18]. 

With regard to the Palestinian population, the Oslo Accords created a complex system in which responsibility for the Palestinian healthcare system was largely transferred to the Palestinian Authority. However, Israel still exerts significant control over access to services, the movement of goods, medicines, and medical personnel. Therefore, Israel remains obligated to ensure the health of the Palestinian population and their access to healthcare resources [19]. 

Israel’s commitment to the provisions of the regulations and the agreement could remove political and other barriers to providing medical assistance to neighboring countries, even in the presence of political or military conflict.

An example of the need for such assistance arose in July 2024, when vaccine-derived type 2 poliovirus was confirmed in sewage samples from Gaza collected in late June 2024, marking the first sign of the virus’s spread in the area. In August 2024, a case of clinical polio was diagnosed in a 10-month-old baby [20]. The State of Israel fulfilled its moral obligation by facilitating the efforts of international organizations to vaccinate 94% of the target population in Gaza against polio during the Iron Swords War, in a campaign described by the WHO as “a remarkable achievement” [21]. 

### Utilitarian aspects

Diseases, especially infectious diseases, do not stop at borders. On July 2024, based on the IHR Emergency Committee advice, the WHO DG declared the monkeypox outbreak as a Public Health Emergency of International Concern (PHEIC). The virus, originating in DRC has been spreading to neighboring countries is threating other countries.

Israel and its neighbors (especially the Palestinian Authority) form a single epidemiological unit. Christian tourists visiting Bethlehem (in the Palestinian Authority) often also visit holy sites in Israel, and workers residing in the Palestinian Authority come daily to work in Israel [18]. Similarly, the above-mentioned polio outbreak in Gaza during the “Iron Swords” war between “Hamas” terror organization and the state of Israel, threatened public health in both Gaza and Israel. Unvaccinated infants, vulnerable populations, soldiers, and hostages were at serious risk of lifelong paralysis [22]. 

The IHR and the ‘Pandemic Agreement’ provisions aimed at ensuring the supply of health products can assist in reducing disease transmission and thus protect not only the population in the location where the outbreak occurred but also others.

Another utilitarian aspect of the IHR and the “Pandemic Agreement” lies in the provisions that promote global collaboration in the development of health products during a pandemic. The lack of collaboration between countries in the field of vaccine development during the COVID-19 pandemic led to a competitive model, where pharmaceutical companies and various nations competed to complete the development of an effective and safe vaccine in the shortest time possible. This competitive model resulted in duplication and inefficiency. The opportunity to conduct joint clinical trials and compare vaccines against each other and placebo in terms of safety, efficacy, benefits, cost, storage, and supply conditions was missed. In Israel, a budget of 230 million NIS ($63.5 million) was allocated to the Biological Institute in Ness Ziona for the development of a vaccine that was not completed [23]. Approximately $800 million was invested in the advance purchase of vaccines from commercial companies– Pfizer, Moderna, and AstraZeneca [24]. 

The obligation of states under the “Pandemic Agreement” to promote the sharing of knowledge relevant to the production of health products, and the establishment of the Pathogen Access and Benefit-Sharing System (PABS) aimed at ensuring the sharing of pathogen samples, could reduce the costs of developing and purchasing vaccines during a future pandemic.^1^

Another aspect that the agreement and regulations will promote is the enhancement of national access to information on global public health threats, which will enable countries to take measures to protect the health of their citizens. In the Israeli context, access to early information about a cholera outbreak following the humanitarian crisis in Syria [26], information about pollution in rivers originating from Lebanon [27], or information about the presence of Poliovirus in Egypt [28] was shared through the IHR and allowed the Israeli health authorities to take steps to mitigate the impact on public health in Israel. Cooperation, which can sometimes be challenging in light of the political conflict, may be facilitated by the IHR and the Pandemic Agreement, which authorize the WHO as an intermediary.

In addition to the utilitarian aspects of global accessibility to health products and valuable information, the provisions of the regulations and the agreement requiring countries to develop capacities for dealing with pandemics at the national level may promote awareness of the need for these capacities in Israel. The Israeli public health services are currently experiencing significant budgetary and staffing deficiencies. This have adversely affected Israel’s preparedness for and response to the COVID-19 pandemic [29]. Notably, the insufficiency of trained personnel to conduct epidemiological investigations necessitated reliance on soldiers lacking expertise in this specialized area [30]. As infection rates surged, the acute shortage of skilled personnel led the Ministry of Health to seek the assistance of the ISA (Israel Security Agency) to support contact tracing efforts [31]. The absence of a structured work plan during the pandemic led to decision-making without consulting experts in fields related to health, such as education, welfare, and economy. Some of the decisions made, aimed at reducing morbidity, disproportionately infringed on individual rights (i.e., the decision to close borders and limit the number of people entering Israel to a quota of only 3,000 per day) [32]. 

Israel’s commitment under the IHR and the Pandemic Agreement to develop capacities for pandemic prevention, preparedness, and response may strengthen national preparedness and promote the allocation of budgets and legislative amendments - which have long been necessary in the field of public health in Israel.

## Conclusions

The IHR and the Pandemic Agreement are designed to enable better responses to future global health threats. At their core lie values of equity and solidarity. The provisions of the regulations and the agreement do not infringe on the sovereignty of member states to make decisions aligned with their national interests but rather aim to establish a mechanism for cooperation.

The WHO policies during the “Iron Swords” war, including its unequivocal support for the Palestinian side and its unwillingness to promote the release of Israeli hostages, have led to Israeli antagonism toward the organization and, as a result, toward the IHR and the Pandemic Agreement.

Nonetheless, despite this sense of distrust, the State of Israel has both ethical commitments and utilitarian interests in advancing the regulations and the agreement. Beyond promoting global and regional health, the regulations and the agreement have the potential to curb the spread of infectious diseases, reduce healthcare costs, ensure access to information on disease outbreaks in neighboring countries, and enhance resource allocation for public health in Israel.

## Data Availability

Not applicable.

## Footnotes

1 The current PABS mechanism within the Pandemic Agreement raises doubts as to whether it will achieve its objectives. The purpose of the mechanism is to regulate access to pathogens and promote collaboration in the development of health products derived from them. However, it creates a situation where developing countries, which often possess the samples, are required to “sell” the pathogens in exchange for future benefits that are concentrated in developed countries and protected by intellectual property rights. To address this inequality, the link between pathogen sharing and receiving benefits must be severed, and the distribution of those benefits must be ensured based on the principle of solidarity [25]. Showcasing successful case studies where pathogen sharing has directly contributed to public health advancements—such as the rapid development of vaccines during global outbreaks—can encourage industrialized nations to engage with PABS. Transparency in the processes of sharing information about pathogens and the benefits derived from it will help prevent corruption and ensure the distribution of benefits based on fairness considerations and in favor of public health. PABS mechanism also raises ethical concerns about the potential misuse of pathogens, such as risks of weaponization or bioterrorism. Without adequate safeguards, these risks pose significant challenges to global security and trust in the framework. To mitigate ethical risks, comprehensive regulatory frameworks are essential, including stringent guidelines for the handling, storage, and transfer of pathogens. Another concern is its potential to create barriers to scientific research. The implementation of strict access regulations can hinder open access to crucial scientific materials, particularly for researchers in low-resource settings, limiting their ability to study pathogens essential for understanding and combating infectious diseases. PABS may also discourage investment in research and development, particularly among smaller pharmaceutical companies, due to the complexity and costs associated with complying with access regulations. This could reduce innovation in the development of vaccines, treatments, and diagnostics. Governments and international organizations can establish funding programs to incentivize research and development related to pathogen access and benefit-sharing. These programs can provide critical support to smaller pharmaceutical companies and research institutions, ensuring sustained innovation.

## Abbreviations

IHR: International Health Regulations
WHO: World Health Organization
WHA: World Health Assembly
ISR: International Sanitary Regulations
PHEIC: Public Health Emergency of International Concern
DG: WHO Director-General
PoE: Points of Entry
SPAR: State Party Annual Reporting
JEE: Joint External Evaluation
IAR, AAR: Intra- and After-Action-Reviews
SIMEX: Simulation Exercises
WGIHR: Working Group on Amendments to the IHR
EB: Executive Board
INB: Intergovernmental Negotiating Body
PABS: Pathogen Access and Benefit-Sharing System
ASPHER: The Association of Schools of Public Health in the European Region
ISA: Israel Security Agency
NPIs: non-pharmaceutical interventions
